# Editorial: Physiology and biochemistry of grain yield potential of rice concerning panicle architectural design

**DOI:** 10.3389/fpls.2024.1349613

**Published:** 2024-02-13

**Authors:** Motohiko Kondo

**Affiliations:** Graduate School of Bioagricultural Sciences, Nagoya University, Nagoya, Japan

**Keywords:** rice, sink, yield, panicle, genetic factor, cultural management

Rice yields have increased significantly since the development of the semi-dwarf IR8 variety in the 1960s due to its widespread use in the world. Semi-dwarf varieties have achieved a dramatic increase in yield potential through both dry matter production and increased harvest index. The former was mainly due to improved light-receiving canopy and enhanced lodging resistance under high nitrogen fertilization, and the latter due to increased panicle size and number of panicles per plant. To further improve yields in the future, the knowledge of genes controlling the number of panicles has increased dramatically to date, and further expansion of sink capacity is feasible. However, to increase yield, the expanded sink needs to be filled, and physiological and genetic factors related to the structure of panicles, translocation of carbohydrates, and source capacity to make the panicle more functional as a sink need to be clarified. It is also important to improve cultivation methods by elucidating factors related to grain filling under various growing environments. In this Research Topic, attempts have been made to elucidate the molecular mechanisms involved in regulating panicle structure and grain ripening at different levels, as well as to examine the effect of cultivation factors, and significant progress is being reported.

Yamamoto et al. focused on the structure of the panicle, which is composed of rachis, primary and secondary branches, and spikelets, and developed a novel model and QTL analysis method to quantify the panicle structural diversity. Using this method, these authors showed that genetic factors are involved in determining panicle structure, in addition to the QTL involved in the number of spikelets, which is already known. This finding is expected to make a significant contribution to panicle design breeding in the future. In particular, it can be used for physiological analysis of why secondary branches are inferior in terms of ripening, which is a major issue for improving yield. Furthermore, using MutMap, the *BOS1* gene was found to encode a new transcription factor involved in the development of the panicle and represent a novel allele of the previously cloned *LAX PANICLE 1* (*LAX1*) gene (Lv et al.). He et al. showed that the *qph12* QTL regulates multiple traits including plant height, panicle length, and spikelet number based on genetic analysis using a population derived from Indica and Japonica. These findings reiterate the close interrelationship between the physiological mechanisms of panicle morphogenesis, plant statue, and canopy structure, and will provide the basis for further elucidation of the molecular mechanisms, including hormone regulation. At the same time, these findings suggest that it may be possible to design the entire plant type while also improving the panicle architecture, and also serve as a cautionary tale that modifications of panicle morphology affect the plant structure. The integrated information of these three reports will help pave the way for precise genetic control of the panicle structure for better grain filling function.

Regarding the agronomic aspect, Wang et al. showed that post-flowering irrigation is effective in reducing losses in grain yield and quality that occur upon water-saving cultivation with drip irrigation. This is a valuable example of a cultivation method that demonstrates the compatibility between water use efficiency and yield. Further studies are expected to elucidate the underlying physiological mechanism.

In summary, the papers in this Research Topic provide a number of significant findings for improving panicle design and enhancing yield potential in rice. Ripening is a complex process determined by multiple factors involving sink capacity, starch synthesis, translocation, and photosynthetic product supply. It seems that what we lack for improving rice yield potential is an understanding of the relationship between panicle morphology and carbon dynamics related to ripening. Learning from the genetic and agronomic factors discussed in this Research Topic, the development of an integrated strategy through further analysis of carbon metabolism may become possible.

## Author contributions

MK: Writing – review & editing.

